# Apple vacuolar sugar transporters regulated by MdDREB2A enhance drought resistance by promoting accumulation of soluble sugars and activating ABA signaling

**DOI:** 10.1093/hr/uhae251

**Published:** 2024-09-03

**Authors:** Lingcheng Zhu, Chunxia Zhang, Nanxiang Yang, Wenjing Cao, Yanzhen Li, Yunjing Peng, Xiaoyu Wei, Baiquan Ma, Fengwang Ma, Yong-Ling Ruan, Mingjun Li

**Affiliations:** State Key Laboratory for Crop Stress Resistance and High-Efficiency Production/Shaanxi Key Laboratory of Apple, College of Horticulture, Northwest A&F University, Yangling, Shaanxi, 712100, China; College of Forestry, Northwest A&F University, Yangling, Shaanxi, 712100, China; State Key Laboratory for Crop Stress Resistance and High-Efficiency Production/Shaanxi Key Laboratory of Apple, College of Horticulture, Northwest A&F University, Yangling, Shaanxi, 712100, China; State Key Laboratory for Crop Stress Resistance and High-Efficiency Production/Shaanxi Key Laboratory of Apple, College of Horticulture, Northwest A&F University, Yangling, Shaanxi, 712100, China; State Key Laboratory for Crop Stress Resistance and High-Efficiency Production/Shaanxi Key Laboratory of Apple, College of Horticulture, Northwest A&F University, Yangling, Shaanxi, 712100, China; State Key Laboratory for Crop Stress Resistance and High-Efficiency Production/Shaanxi Key Laboratory of Apple, College of Horticulture, Northwest A&F University, Yangling, Shaanxi, 712100, China; State Key Laboratory for Crop Stress Resistance and High-Efficiency Production/Shaanxi Key Laboratory of Apple, College of Horticulture, Northwest A&F University, Yangling, Shaanxi, 712100, China; State Key Laboratory for Crop Stress Resistance and High-Efficiency Production/Shaanxi Key Laboratory of Apple, College of Horticulture, Northwest A&F University, Yangling, Shaanxi, 712100, China; State Key Laboratory for Crop Stress Resistance and High-Efficiency Production/Shaanxi Key Laboratory of Apple, College of Horticulture, Northwest A&F University, Yangling, Shaanxi, 712100, China; State Key Laboratory for Crop Stress Resistance and High-Efficiency Production/Shaanxi Key Laboratory of Apple, College of Horticulture, Northwest A&F University, Yangling, Shaanxi, 712100, China; Division of Plant Sciences, Research School of Biology, The Australian National University, Canberra, ACT 2601, Australia; State Key Laboratory for Crop Stress Resistance and High-Efficiency Production/Shaanxi Key Laboratory of Apple, College of Horticulture, Northwest A&F University, Yangling, Shaanxi, 712100, China

## Abstract

Soluble sugars are not only an important contributor to fruit quality, but also serve as the osmotic regulators in response to abiotic stresses. Early drought stress promotes sugar accumulation, while specific sugar transporters govern the cellular distribution of the sugars. Here, we show that apple plantlets accumulate soluble sugars in leaf tissues under drought stress. Transcriptional profiling of stressed and control plantlets revealed differential expression of several plasma membrane—or vacuolar membrane-localized sugar transporter genes. Among these, four previously identified vacuolar sugar transporter (VST) genes (*MdERDL6–1*, *MdERDL6–2*, *MdTST1*, and *MdTST2*) showed higher expression under drought, suggesting their roles in response to drought stress. Promoter *cis*-elements analyses, yeast one-hybrid, and dual-luciferase tests confirmed that the drought-induced transcription factor MdDREB2A could promote the expression of *MdERDL6–1/−2* and *MdTST1/2* by binding to their promoter regions. Moreover, overexpressing of each of these four *MdVSTs* alone in transgenic apple or *Arabidopsis* plants accumulated more soluble sugars and abscisic acid (ABA), and enhanced drought resistance. Furthermore, apple plants overexpressing *MdERDL6–1* also showed reduced water potential, facilitated stomatal closure, and reactive oxygen species scavenging under drought conditions compared to control plants. Overall, our results suggest a potential strategy to enhance drought resistance and sugar accumulation in fruits through manipulating the genes involved in vacuolar sugar transport.

## Introduction

Crop yield and quality can be severely impacted by drought, and the occurrence of drought conditions worldwide may be exacerbated by climate change [1, 2]. Drought stress refers to prolonged conditions of water deficit, when soil moisture is insufficient to support transpiration and photosynthesis. Drought induces hyperosmotic stress in plant cell, resulting in an overabundance of ROS (reactive oxygen species), harm to cellular structures and compartments, accelerated protein degradation, and cell death [3].

Terrestrial plants have evolved a variety of biological strategies to withstand the challenges posed by drought. For example, through a process called osmotic adjustment, cells increase the content of soluble sugars, proline, and glycine betaine, which protect cellular molecules and structures against dehydration [4, 5]. Soluble sugars are the predominant organic solutes responsible for osmotic adjustment in plants under drought stress. Increasing soluble sugar content also helps to maintain turgor [6].

Translocation and accumulation of soluble sugar is tightly controlled by sugar transporters localized on the cell membrane and vacuolar membrane. Sugar transporter families are classified according to substrate characteristics and localization, and include SUT (sucrose transporter), MST (monosaccharide transporter), and the latest identified SWEET (sugars will eventually be exported transporter) families [7]. Of these transporters, ERDL6 (early response to dehydration six-like transporter), TST (tonoplast sugar transporter), and VGT (vacuolar glucose transporter) from MST family, SUT4 homologs, and the clade IV members of SWEET, are only reported as vacuolar sugar transporters (VSTs) [8, 9].

The gene expression and protein activity of sugar transporters can respond to various abiotic stresses. Three sucrose transporter genes from *Arabidopsis thaliana* (*Arabidopsis*), *AtSUC2/4/9*, play important roles in resisting drought, high salinity, and low temperature at whole-plant level [10, 11]. In apple, tonoplast-localized MdSUT2 is phosphorylated by the CBL-interacting protein kinases MdCIPK13 and MdCIPK22 to increase salt and drought resistance, respectively [12, 13]. The tonoplast sugar transporter genes *AtTST1/2* mediate cold temperature-induced accumulation of hexose in the vacuoles in *Arabidopsis* [14]. In sugar beet, the vernalization-mediated sink-to-source change of the taproot is associated with changes in expression of the *BvTST2.1* and *BvSUT4* genes, which are crucial for vacuolar sucrose import and export [15]. In *Arabidopsis*, the *EARLY RESPONSIVE TO DEHYDRATION* homologs, *ERD6*, is rapidly induced upon dehydration, which encodes a likely sugar transporter [16]. In addition, transgenic overexpression of the homologous *AtERDL6*, encoding a vacuolar glucose exporter, resulted in decreased accumulation of monosaccharides and a decrease in cold tolerance [17, 18]. The sucrose transporter genes *AtSWEET11/12* play vital roles in phloem loading, and can rapidly be phosphorylated by the protein kinases AtSnRK2.2/2.3/2.6 to enhance sucrose transport activity and enhance root growth under drought conditions [19]. Transgenic overexpression of *AtSWEET16* conferred higher tolerance to freezing, likely by increasing the vacuolar glucose and sucrose levels [20]. In contrast, knock-out mutants of *AtSWEET17* showed altered sugar distribution and reduce lateral root growth, associated with impaired drought resistance [21].

**Figure 1:**
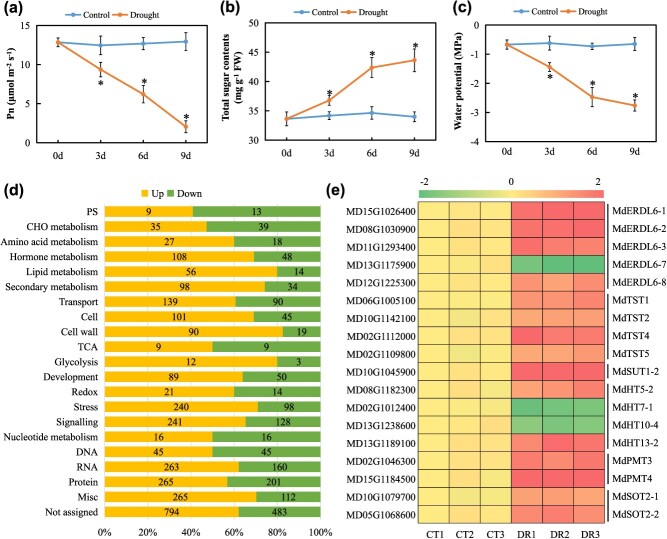
The photosynthesis rate, total sugar content, water potential, and identification of DEGs in apple leaves under drought conditions. (**a-c**) Variations of Pn, total soluble sugar contents, and water potential in apple leaves at 0, 3, 6, and 9 DAT. The bars represent mean values ± SD (*n* = 5). ^*^P < 0.05 indicates significant difference relative to the well-watered control. (**d**) Functional category enrichment (modified MapMan bins) of DEGs in leaves from drought-treated plants or well-watered controls, at 6 DAT (see Supplementary Excel 1 for details). Orange and green colors indicate significantly upregulated and downregulated genes, respectively. (**e**) A heat map illustrating all differentially expressed sugar transporter genes in the apple leaves of drought at 6 DAT compared with the well-watered controls. The differential expression represents the log2 values, with the FPKM in CT1 set as 1 for each gene. CT, well-watered control; DR, drought treatment.

The DREB transcription factors (dehydration-responsive element-binding proteins) are integral in managing responses to abiotic stress [22]. In *Arabidopsis*, *AtDREB1A* is induced by low temperatures, whereas *AtDREB2A* is induced by drought [23]. The DREB2A positively regulates the drought-responsive genes *RD29A* and *RD29B* via interacting with the DRE element in their promoters, thereby enhancing drought tolerance [24]. Recent studies showed that DREB2 mediated soluble sugar change in apple and tomato plant tissues under drought stress [25, 26], but the underlying mechanisms under stress remain to be elucidated.

Potential strategies for concurrent improvement of abiotic stress resistance and fruit quality are highly desired for horticultural crop breeding. We have previously identified two classes of VSTs, *MdERDL6–1/−2* and *MdTST1/2*, that positively regulated soluble sugar accumulation in apple fruits [27, 28]. In this study, we demonstrated that overexpression of these four *MdVSTs* alone significantly enhanced drought resistance, and the *MdDREB2A-MdVSTs* regulatory module might be a potential approach to improve apple varieties. Overall, our study provides theoretical strategy for simultaneous breeding for new cultivars with improved drought tolerance and fruit quality.

## Results

### Drought stress induces sugar accumulation and decreases water potential in apple leaves

This study utilized the popular ‘GL3’ genotype, a clonal material with a pure genetic background selected from seedlings of ‘Royal Gala’ apple based on its enhanced efficiency for *Agrobacterium*-mediate transformation and regeneration [29]. Two groups of ‘GL3’ plantlets were divided, one group was subjected to water-deficit stress conditions, whereas the other group received continuous irrigation to keep the soil water content saturated (control). Then, we determined the net photosynthesis rate, total sugar content, and water potential in leaves of 2-month-old ‘GL3’ plants at 0, 3, 6, and 9 days after the onset of drought treatment (DAT). Results showed that photosynthetic rate declined significantly and continuously throughout this period, reaching nearly undetectable levels by 9 DAT (Fig. 1a). In contrast, the total sugar content in leaves gradually and significantly accumulated over this period, showing an increase of ~28% compared to the well-watered control by 9 DAT (Fig. 1b). Consistent with the increase in sugar content, leaf water potential decreased throughout the drought treatment (Fig. 1c).

### Drought-induced MdDREB2A regulates the expression of vacuolar sugar transporter genes

To explore molecular basis of the observed increase in leaf-soluble sugars under drought treatment, this work carried out RNA-seq analysis of apple leaves collected at 6 DAT from stressed or control plants, and identified genes that were differentially expressed. In total, 4562 differentially expressed genes (DEGs) were identified; 2923 were expressed more strongly in the stressed plants, while 1639 were expressed more strongly in the controls (Supplementary Excel 1). Functional enrichment analysis showed that these DEGs clustered in 21 functional groups (annotated with MapMan), including hormone metabolism, transport, signaling, RNA, and protein (Fig. 1d).

Since sugar transporters, and especially VSTs, control sugar accumulation in plant cells [27], we identified putative sugar transporter genes from the set of DEGs in the ‘transport’ functional cluster. Nine of the 18 differentially expressed sugar transporter genes that were found (Fig. 1e), comprising homologs of *ERDL6* and *TST*, were predicted to be located on the tonoplast, while the remainder were anticipated to reside on the cell membrane [9]. Notably, four VST genes, *MdERDL6–1*, *MdERDL6–2*, *MdTST1,* and *MdTST2*, exhibited the highest expression abundances than other transporters under drought conditions (Supplementary Excel 1), indicating that they may play a more important role in responding to drought. Based on these observations, these four VST genes were subjected to further functional evaluations.

**Figure 2:**
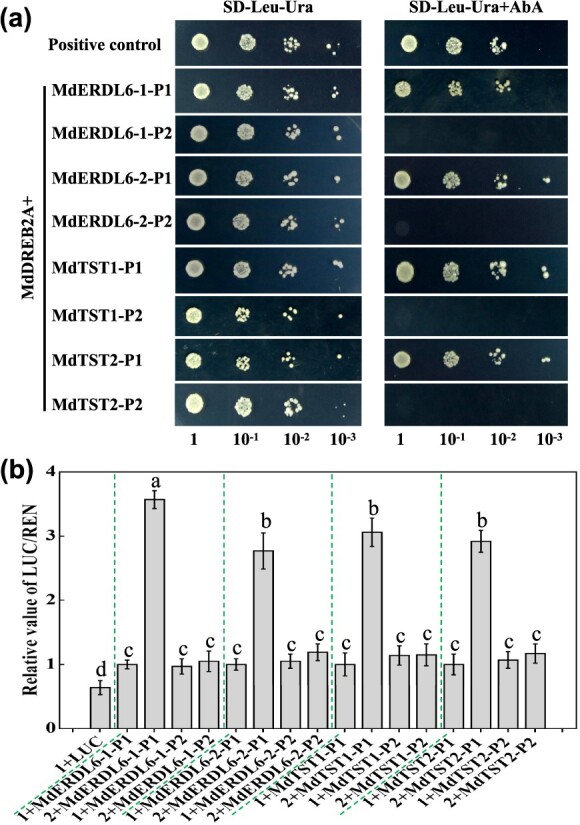
The regulatory relationship between MdDREB2A and four VST genes from apple. (**a**) Y1H assays demonstrating the binding capacity of MdDREB2A to the truncated promoters (P1 and P2) of *MdERDL6–1/−2* and *MdTST1/2* on SD-Leu-Ura medium plus AbA. The screening AbA concentrations of *MdERDL6–1/−2-* and *MdTST1/2-*truncated promoters were 200, 300, 200, and 450 ng/ml, respectively. pAbAi-P53 and pGADT7–53 were used as positive controls. P1 promoter regions contain a DRE or GCC-box element, while P2 promoter regions do not. (**b**) LUC assays of MdDREB2A and the truncated promoters (P1 and P2) of *MdERDL6–1/−2* and *MdTST1/2*. ‘1’ represents the empty vector pGreenII 62SK; ‘2’ represents 35S:MdDREB2A. The negative control is the combination of pGreenII 62SK + pGreenII 0800LUC. The luciferase values in the combinations of pGreenII 62SK + MdVSTs-P1 were set as 1. The bars represent mean values ± SD (*n* = 3). Different letters indicate significant differences as determined by one-way ANOVA (with Tukey’s test) (*P* < 0.05).

With the observation that *MdERDL6–1/−2* and *MdTST1/2* showed similar expression patterns that gradually increased with fruit development [27] and were all induced under drought stress (Fig. 1e), we hypothesized that all four genes may be under common regulatory control. The promoters of these four genes contain DRE or GCC-box elements, which could be bound by the DREB transcription factor [24]. Our transcriptional data exhibited that *MdDREB2A* was expressed to significantly higher levels in apples under drought stress (Fig. S1). Also, recent studies showed that DREB2 mediated soluble sugar accumulation in plant tissues of apple and tomato under drought stress [25, 26]. Hence, we hypothesize that whether MdDREB2A could regulate the *MdVSTs* expression. To test this, we carried out yeast one-hybrid (Y1H) and dual-luciferase (LUC) tests using truncations of the *MdERDL6–1/−2* and *MdTST1/2* promoters, designated P1 (with DRE or GCC-box) and P2 (without DRE or GCC-box) (Fig. S2). *In vitro*, we found that transformants with the P1 promoters and pGADT7-MdDREB2A thrived on SD-Leu-Ura medium containing Aureobasidin A (AbA), whereas those harboring P2 promoters failed to grow (Fig. 2a). Moreover, co-expression of all P1:LUC constructions with 35S:MdDREB2A significantly increased luciferase activities, whereas co-expression of all P2:LUC constructions with 35S:MdDREB2A did not result in increased luciferase activities (Fig. 2b). In summary, these findings suggest that MdDREB2A activates the transcript of *MdERDL6–1/−2* and *MdTST1/2* via interacting with the DRE or GCC-box elements on their promoters.

### Expression of *MdERDL6–1/−2* and *MdTST1/2* in apple and *Arabidopsis* enhances drought resistance

**Figure 3:**
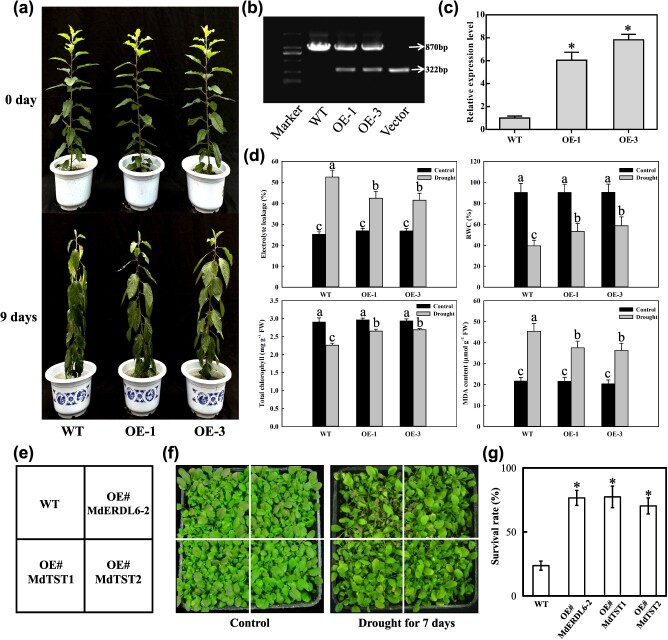
Overexpression of *MdERDL6–1/−2* and *MdTST1/2* alone promotes drought resistance. (**a**) Phenotypes of non-transgenic and two *MdERDL6–1*-overexpressing apple lines prior to and after 9 days of drought. (**b**) Verification of the transgenic apple lines using PCR. The upper band represents an 870-bp DNA fragment from the genomic copy of *MdERDL6–1* across introns 9–12, while the lower band represents the corresponding 322-bp DNA fragment from the introduced *MdERDL6–1* cDNA-lacking introns. (**c**) Relative expression of *MdERDL6–1* mRNA in transgenic apples. The bars represent mean values ± SD (*n* = 3). *P < 0.05 indicates significant difference relative to the well-watered control. **(d)** Electrolyte leakage, RWC, total chlorophyll concentration, and MDA concentration in WT and *MdERDL6–1*-overexpressing apple lines under control or drought conditions at 9 DAT. The bars represent mean values ± SD (*n* = 3). Different letters indicate significant differences as determined by one-way ANOVA (with Tukey’s test) (*P* < 0.05). **(e)** Planting map of WT and overexpression of *MdERDL6–2*, *MdTST1*, and *MdTST2* transgenic *Arabidopsis*. **(f)** Phenotypes of WT and transgenic *Arabidopsis* under well-watered control and drought treatment for 7 days. **(g)** Survival rate of WT and transgenic *Arabidopsis* after drought treatment. The bars represent the mean value ± SD (*n* = 3). *P < 0.05 indicates a significant difference from WT.

Expression analysis by RT-qPCR revealed that *MdERDL6–1/−2* and *MdTST1/2* were expressed in multiple apple structures, including growing shoot tips, roots, flowers, young and old leaves, and young and mature fruits (Fig. S3). To evaluate a potential role for these genes in drought resistance, transgenic apple plants overexpressing *MdERDL6–1* and *Arabidopsis* expressing *MdERDL6–2* or *MdTST1/2* were obtained and evaluated under stress or control conditions. There was no obvious effect of expression of any of the genes in the transgenic lines at well-watered conditions (Fig. 3a–c, e, f). However, after 7 days of drought treatment, *Arabidopsis* plants expressing *MdERDL6–2* or *MdTST1/2* showed higher survival rates than wild type (WT) (Fig. 3f, g). Similarly, transgenic apple plants overexpressing *MdERDL6–1* showed only slight wilting after 9 days of drought, whereas control plants wilted severely (Fig. 3a). Under non-stress controls, no notable distinctions were observed between *MdERDL6–1* and non-transgenic controls for electrolyte leakage, relative water content (RWC), total chlorophyll concentrations, or malondialdehyde (MDA) content. In contrast, after 9 days of drought, *MdERDL6–1* leaves exhibited lower levels of electrolyte leakage and MDA, but higher RWC and total chlorophyll, compared with the WT (Fig. 3d), which suggested that *MdERDL6–1* overexpression apple plants enhanced drought resistance.

To determine if *MdERDL6–1* overexpression altered cellulars sugar homeostasis, we next evaluated transcript level of *MdCAB1*, which is a sugar-sensitive gene and is negatively regulated by cytosolic glucose level [18, 30]. The results showed that transgenic plants overexpressing *MdERDL6–1* showed decreased *CAB1* expression compared with the WT under non-stress and drought conditions, whereas drought conditions decreased *CAB1* expression further (Fig. S4). This is consistent with increased transport of glucose from the vacuole to cytosol associated both with drought stress and with *MdERDL6–1*.

### *MdERDL6–1/−2* and *MdTST1/2* overexpression induces sugar accumulation under drought stress

**Figure 4:**
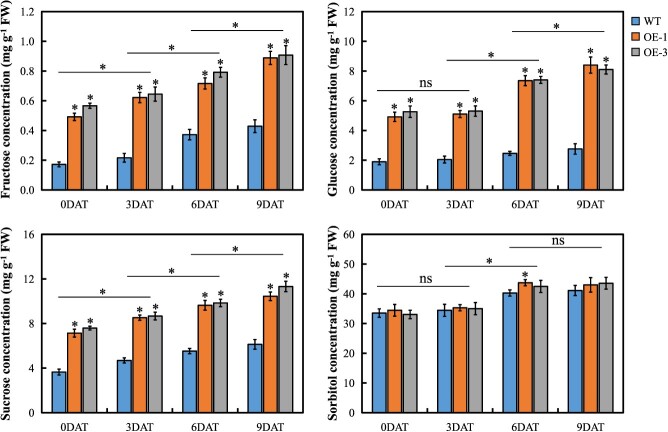
Variations in the concentrations of sugars and sorbitol in leaves of WT and *MdERDL6–1*-overexpressing plants under control or drought conditions. The bars represent the mean values ± SD (*n* = 3). ^*^P < 0.05 indicates significant difference relative to the control. ns, not significant.

Transgenic expression of *MdERDL6–1/−2* or *MdTST1/2* in apple and *Arabidopsis* significantly increased sugar accumulation under well-watered and drought conditions (Figs. 4 and S5). In well-watered apple control plants, overexpression of *MdERDL6–1* was associated with increasing fructose, glucose, and sucrose, whereas no significant difference was determined in sorbitol concentration. During drought treatment, the contents of glucose, fructose, and sucrose showed continuous significant increases in both the WT and *MdERDL6–*1-overexpressing lines, while sorbitol concentration increased significantly only at 6 DAT (Fig. 4). Our previous reports showed that *MdERDL6–1* is coordinately expressed with *MdTST1/2* [27, 28]. Therefore, the expression of *MdTST1/2* was also evaluated in *MdERDL6–1-*overexpressing apple plants, and the result demonstrated that the *MdTST1/2* expression was notably enhanced in the transgenic apple plants, regardless of whether under control or drought conditions. (Fig. S6).

**Figure 5:**
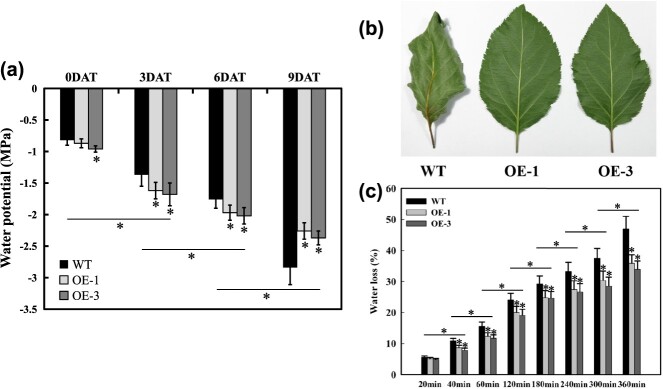
Effects of *MdERDL6–1* overexpression on the water potential and water loss rate in apple leaves. (**a**) The water potential in apple leaves of WT and *MdERDL6–1* overexpression apples after 0, 3, 6, and 9 days of drought. (**b**) Representative images showing water loss for 360 min in detached WT and *MdERDL6–1* overexpression apple leaves. (**c**) The rates of water loss in detached leaves from both WT and *MdERDL6–1* overexpression plants at 20, 40, 60, 120, 180, 240, 300, and 360 min. Bars represent the mean values ± SD (*n* = 3). ^*^P < 0.05 indicates significant difference relative to the control treatment.

Moreover, water potential, which is affected by soluble sugar levels, was analyzed in leaves of non-stress and drought-treated plants. Water potential in two *MdERDL6–1* transgenic leaves was discovered to be reduced compared to the WT prior to drought treatment, while both lines showed significantly lower water potential at 3 and 6 DAT (Fig. 5a). In contrast, at 9 DAT, both transgenic lines showed higher water potential than the control, potentially due to loss of leaf function in the WT leaves by this point. This result was also supported by measurements of the rate of water loss in leaves. No notable variation in water loss was seen between leaves from the WT and *MdERDL6–1-*overexpressing plants placed on a ventilated lab bench for 20 min. In contrast, the transgenic plants lost water at a slower rate than WT from 40 to 360 min (Fig. 5c). WT leaves showed severe wilting after 6 h, leaves from *MdERDL6–1*-overexpressing lines were relatively unaffected (Fig. 5b, c). Taken together, these findings suggest that increased soluble sugar concentrations in the *MdERDL6–1*-overexpressing leaves maintained normal moisture status by decreasing water potential and limiting water loss.

**Figure 6:**
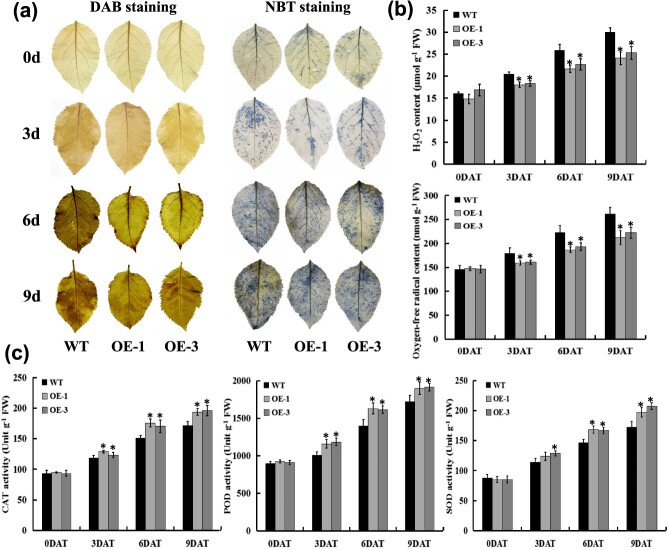
Effects of *MdERDL6–1* overexpression on ROS level and its scavenging enzyme activities. (**a**) The representative staining images of leaves showing H_2_O_2_ and O_2_^−^ accumulation under drought stress. (**b**) H_2_O_2_ and O_2_^−^ contents in the WT and *MdERDL6–1*-overexpressing apple leaves under drought stress. (**c**) The activities of CAT, POD, and SOD in the WT and *MdERDL6–1*-overexpressing apple leaves under drought stress. The bars represent mean values ± SD (*n* = 3). ^*^P < 0.05 indicates significant difference relative to the control treatment.

### *MdERDL6–1* overexpression in apple enhances photosynthesis efficiency under drought stress

To assess the impact of *MdERDL6–1* overexpression on photosynthesis under drought stress, we measured photosynthesis rate (Pn), transpiration rate (Tr), and variable/maximal chlorophyll fluorescence (Fv/Fm) in WT and *MdERDL6–1*-overexpressing leaves under non-stress and drought conditions (Fig. S7). In the absence of drought stress, there were no obvious differences in these photosynthesis parameters when comparing the lines overexpressing *MdERDL6–1* with the WT plants. However, *MdERDL6–1*-overexpressing lines maintained consistently higher Pn values than those of WT plants from 3 to 9 DAT (Fig. S7a). *MdERDL6–1*-overexpressing lines also showed significantly lower Tr rates at 3 and 6 DAT (Fig. S7b). Moreover, *MdERDL6–1*-overexpressing lines showed consistently higher Fv/Fm values in comparison to WT lines under drought stress (Fig. S7c, d), indicating reduced damage to photosystem II (PSII) in the transgenic apples under drought.

### *MdERDL6–1* overexpression in apple accelerates ROS-scavenging under drought stress

To evaluate the potential influence of *MdERDL6–*1 overexpression on ROS accumulation, leaves from *MdERDL6–1*-overexpressing and WT plants were collected at 0, 3, 6, and 9 DAT for hydrogen peroxide (H_2_O_2_) and superoxide anion (O_2_^−^) detection by diaminobenzidine (DAB) and nitro blue tetrazolium (NBT) staining, respectively. Results were consistent with a gradual accumulation of ROS during the drought treatment for both the WT and *MdERDL6–1* overexpression apples (Fig. 6a). However, the staining intensity of both DAB and NBT was generally decreased in the *MdERDL6–1* overexpression leaves compared with the WT at 3 DAT and thereafter. In addition, the continual rise in H_2_O_2_ and O_2_^−^ during the drought stress was decreased in the *MdERDL6–1*-overexpressing plants relative to WT (Fig. 6b), consistent with the ROS staining of the leaves. Furthermore, we also measured the ROS concentrations in leaves using 2′,7′-dichlorofluorescin diacetate (H_2_DCFDA), an oxidation-sensitive fluorescence probe. The results showed that drought stress increased the accumulation of ROS fluorescence in both WT and *MdERDL6–1*-overexpressing leaves, but *MdERDL6–1*-overexpressing plants accumulated less ROS than that of WT (Fig. S8). These observations were consistent with the above measurements.

To explain the observed decrease in ROS accumulation in leaves of the transgenic lines, we analyzed ROS-scavenging enzyme activity. Results showed that the catalase (CAT), peroxidase (POD), and superoxide dismutase (SOD) activities did not show a significant variation between the apple plants overexpressing *MdERDL6–1* and the WT plants at 0 DAT. However, the continual rise in activity for these enzymes during the drought stress was increased in the *MdERDL6–1*-overexpressing plants relative to WT (Fig. 6c). This suggests that *MdERDL6–1* may participate in activating the ROS system during the initial phase of drought treatment, and that the increased ROS-scavenging activity may contribute to the enhanced drought tolerance observed for *MdERDL6–1*-overexpressing lines.

### *MdERDL6–1* overexpression in apple promotes ABA synthesis and signaling under drought stress

**Figure 7:**
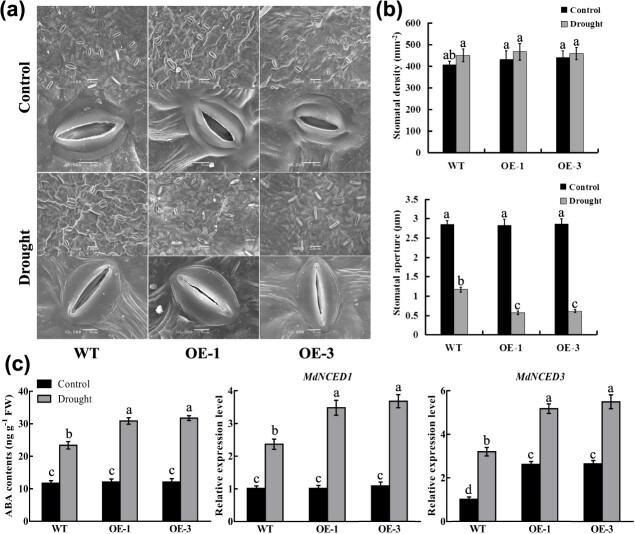
Effects of *MdERDL6–1* overexpression on the stomatal profiles and ABA contents in apple leaves. (**a**) The images of stomatal morphology. The scale bars in the upper and lower images represent 20 and 5 μm, respectively. (**b**) The stomatal density and stomatal aperture in WT and *MdERDL6–1* overexpression apple leaves under normal and drought. (**c**) The content of ABA and expression of *MdNCED1/3* in WT and *MdERDL6–1* overexpression apple leaves under normal and drought stress. The bars represent mean values ± SD (*n* = 3). Different letters indicate significant differences as determined by one-way ANOVA (with Tukey’s test) (*P* < 0.05).

As described above, the transpiration rate for *MdERDL6–1* transgenic apple leaves was notably reduced compared to the WT at 3 and 6 days after drought treatment (Fig. S7b), which could potentially be attributed to changes in stomatal parameters. The stomatal guard cell is a critical regulator of plant water budget during drought. Thus, we analyzed leaf stomatal structure using scanning electron microscopy. Under non-drought conditions, no variation was observed in stomatal density and aperture between the *MdERDL6–1* overexpression and WT lines (Fig. 7a, b). After 9 days of drought stress, stomatal apertures decreased for all plants, but stomatal apertures for the transgenics were significantly less than WT. Stomatal density was not affected either by drought or by overexpression of *MdERDL6–1* (Fig. 7a, b). These results indicated that overexpressing *MdERDL6–1* in apple promoted stomatal closure during drought treatment.

We then determined the abscisic acid (ABA) content in apple leaves, which is a crucial hormone involved in regulation of stomatal closure. As anticipated, drought treatment increased ABA levels in both WT and *MdERDL6–1* overexpressing leaves, and ABA content was significantly higher in transgenic plants by 9 DAT, which was consistent with the observations of stomatal aperture. While no ABA difference was determined between *MdERDL6–1* overexpression and WT lines under the well-watered conditions (Fig. 7c). Importantly, the enhanced ABA contents were also confirmed in *MdERDL6–2* or *MdTST1/2* overexpressing *Arabidopsis* lines under drought stress (Fig. S9), indicating that these four *MdVSTs* might share similar molecular mechanism in response to water-deficient environment. Moreover, in transgenic apple plants, two ABA biosynthetic genes *MdNCED1* and *MdNCED3*, significantly increased after drought stress (Fig. 7c), with larger increases seen in the transgenic lines. These findings suggest that *MdERDL6–1* promotes ABA accumulation by upregulating the *NCED* expression under drought conditions.

ABA-involved drought resistance in plant is mediated mainly by members of the AREB/ABF (ABRE-binding protein/factor) transcription factor subfamily, which belongs to Clade A of the bZIP (basic leucine zipper) family [31]. The apple genome encodes five members of *MdAREB* family [32]. To determine the influence of *MdERDL6–1* on the expression of these genes, this study conducted an analysis comparing the gene expression characteristics between *MdERDL6–1*-overexpressing and WT plants. RT-qPCR results indicated that *MdAREB1.1/1.2/1.3* exhibited higher expression in the *MdERDL6–1*-overexpressing plants than those in WT lines under drought conditions. Consistently, expression level of *MdSnRK2.6*, which is a homolog of *OST1* (open stomata 1) that controlled stomatal closure, showed similar accumulation patterns in the *MdERDL6–1*-overexpressing apples (Fig. S10). Taken together, these findings suggest that *MdERDL6–1* overexpression activates ABA signal transduction and promotes stomatal closure under drought stress.

## Discussion

### *MdDREB2A* regulates vacuolar sugar transporters under drought stress

Drought is typically accompanied with osmotic stress in plants, leading to increased synthesis and accumulation of osmolytes [1, 2]. As an osmotic regulator, soluble sugars accumulate rapidly upon cellular stress to avoid cellular damage caused by water deficit. In this study, drought treatment inhibited the photosynthesis rate, increased sugar contents, and decreased water potential in the leaves of apple plants (Fig. 1a–c). The increase in sugar contents in the leaves under low-photosynthesis conditions can be explained by the following three aspects: (i) drought stress significantly induces the expression of the sugar transporter genes (Fig. 1), which are responsible for transporting sugars from the cell space into the cytoplasm or from the cytoplasm into the vacuoles; (ii) the low concentration of sugars in the cytoplasm induces the expression of genes encoding amylase (Supplementary Excel 2), converting starch into sugars and transferring them into the cytoplasm to replenish the low sugar concentration there; and (iii) the increase in the relative content of dry matter due to water loss in leaves under drought conditions. Notably, raffinose is considered to play crucial roles in plant drought tolerance based on its accumulation in leaves when plants encounter drought stress [33]. Consistent with this, we found that several raffinose synthesis-related genes were differentially expressed (Supplementary Excel 2).

Given that vacuoles occupy substantial proportion of cellular space and are the predominant storage site of soluble sugars in plant cells [27], we focused on VSTs in this study. DREB2A functions as a pivotal transcription factor, orchestrating drought tolerance in plants by modulating several drought-related structural genes, such as *KIN1*, *RD17/19A*, and *COR15A/B* [24]. This study identified MdDREB2A as an upstream transcriptional activator of *MdERDL6–1/−2* and *MdTST1/2* (Fig. 2). As far as we are aware, this study presents the initial evidence that *VSTs* serve as the downstream target genes of DREB2A.

### Vacuolar sugar transporters promote drought resistance in transgenic plants

In *Arabidopsis*, *AtERDL6* and *AtTST* members have been proved to be induced by a range of abiotic stressors [14, 16–18, 34]. Although most horticultural plant researches have demonstrated that ERDL6 and TST homologous genes exhibit a substantial positive correlation with sugar accumulation in fruits [7, 27], their roles in plant abiotic stress responses remain unknown. Our finding was that overexpression of *MdERDL6–1/−2* or *MdTST1/2* alone significantly increased drought resistance in *Arabidopsis* and apple. This outcome aligns with the discovery that tonoplast-localized *AtSWEET17* is activated under drought conditions, with its mutants displaying impaired drought tolerance (Fig. 3) [21]. Overexpression of *MdSWEET17* resulted in significant accumulation of fructose and improved drought resistance in tomato plants [35]. Collectively, these findings provide an unprecedented strategy for enhanced drought resistance in plants by increasing *VST* expression.

### Overexpression of *MdERDL6–1* induces sugar and ABA accumulation under drought stress

Subcellular soluble sugar levels can influence drought resistance in plants. Under drought conditions, *MdERDL6–1* overexpression increased cytosolic glucose content, and synchronously elevated the vacuolar concentrations of sucrose, fructose, and glucose by activating the tonoplast sugar importer genes *MdTST1/2*. These effects resulted in a drastic decrease in leaf water potential of transgenic apples (Fig. 5a). High concentrations of sugars have been predicted to directly scavenge ROS [36], and evaluation of ROS levels in this study showed that *MdERDL6–1*-overexpressing plants accumulated less H_2_O_2_ and O_2_^−^, which could be attributed to significantly increased ROS-scavenging enzyme activities (Figs. 6 and S8). Further assessment of photosynthetic efficiency in the transgenic plants revealed that despite showing lower Tr at 3–6 DAT, increasingly higher Pn value was observed (Fig. S7a, b), which suggested that decreased water loss maintained the normal physiological status of the transgenic plants.

Several studies have suggested that crosstalk between sugars and ABA is crucial for plant growth and fruit ripening. For example, the sugar-insensitive mutants *sis4*/*5* exhibit deficient ABA synthesis and transduction in *Arabidopsis* [37]. Similarly, ABA-deficient *Arabidopsis* mutants *aba1–1/2–1/3–2* are also insensitive to glucose [38]. In addition, sucrose acts as a signal molecule to accelerate fruit ripening in strawberry by inducing ABA synthesis [39]. In this work, overexpression of *MdERDL6–1* activated the ABA synthesis genes *MdNCED1*/*3* thereby increasing ABA under drought (Fig. 7c). Consequently, increased ABA synthesis elevated the expression of *MdSnRK2.6*, which functions as an enhancer of ABA-mediated stomatal closure [40], and decreased leaf water loss. Moreover, activation of ABA signaling by AREB members, such as *MdAREB1.1/1.2/1.3*, was detected in the transgenic apple plants (Fig. S10). Overall, sugar-induced ABA signal transduction might be a factor driving drought resistance. However, the mechanism of crosstalk between sugar and ABA in apple leaves remains to be further documented.

### A potential strategy for simultaneous improvement of abiotic stress resistance and fruit quality in horticultural crops

**Figure 8:**
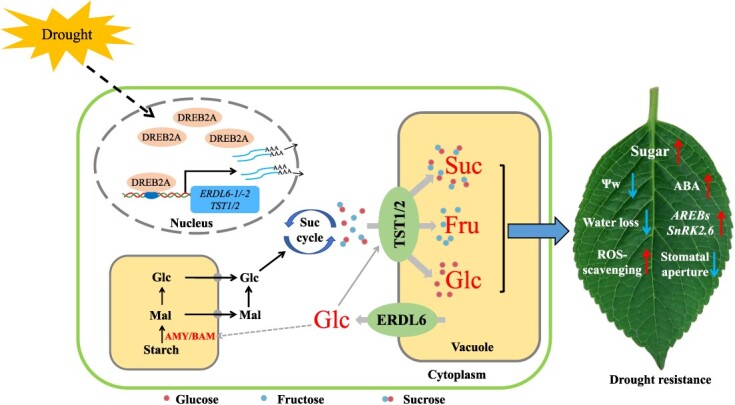
A diagram that depicts how VSTs manage the drought resistance in apple plants. Drought stress leads to increased expression of *MdDREB2A*, which promotes expression of four VST genes, *MdERDL6–1/−2* and *MdTST1/2*, changing sugar homeostasis across the vacuolar membrane. In addition, on the one hand, *MdERDL6–1*-mediated cytosolic glucose efflux might induce the expression of amylase genes, converting starch into soluble sugars and transferring them into the cytoplasm; on the other hand, *MdERDL6–1*-mediated cytosolic glucose signaling also activated *MdTST1/2* transcript and increased sugar accumulation into vacuoles, leading to increased resistance to drought stress by simultaneously decreasing water potential (Ψw) and rate of water loss, accelerating ROS-scavenging activities, increasing ABA synthesis, and enhancing both stomatal closure and ABA signal transduction. Glc, glucose; Fru, fructose; Suc, sucrose; Mal, maltose.

Increasing soluble sugars can improve the flavor and quality in fruits, while in leaves can enhance abiotic stress resistance. Our previous studies revealed that overexpression of *ERDL6–1*, *TST1*, *TST2*, and *HT2.2* from apple significantly increased sugar concentration in transgenic plants [28, 41, 42]. In this study, we discovered that these genes or their paralogs could also respond to drought stress, with apple plants overexpressing *MdERDL6–1* exhibiting enhanced drought resistance, likely due to dramatically increased sugar accumulation and decreased water potential in leaves (Fig. 8). Similarly, transgenic overexpression of an ABA-induced tonoplast sucrose transporter gene, *MdSUT2*, which is regulated by the transcription factor MdAREB2, led to a heightened buildup of sucrose and bolstered drought tolerance in apple plants [12, 32]. Therefore, we propose that manipulation of the VST genes represents an unprecedented strategy for potential simultaneous improvement of drought resistance and fruit quality in horticultural plants.

## Materials and methods

### Plant materials and treatments

Plants of the 'GL3' variety, aged 2 months, were cultivated in plastic containers filled with a blend of soil, sand, and organic fertilizer (3:1:1, v:v:v), and subsequently categorized into two distinct groups. One cohort of plants was exposed to drought stress, whereas the other was consistently watered to preserve soil moisture at a saturated level, serving as the control group. Photosynthesis rates, sugar contents, and water potential in leaves exposed to drought were measured at 0, 3, 6, 9 days post-treatment initiation. In addition, the leaves of well-watered and drought-treated plants were collected at 6 DAT for RNA-seq analysis to screen for DEGs.

WT and *MdERDL6–1* overexpression ‘GL3’ apples, propagated through tissue culture, were cultivated on an MS (Murashige and Skoog) medium supplemented with 0.3 mg l^−1^ 6-BA (6-benzylaminopurine) and 0.2 mg l^−1^ IAA (indoleacetic acid). Plants were later rooted on MS medium enriched with 0.5 mg l^−1^ IBA (indolebutyric acid) and 0.5 mg l^−1^ IAA, maintained at a temperature of 23°C with a photoperiod of 16 h of light and 8 h of darkness, over a period of 45 days. After rooting, plants were moved into plastic pots filled with a blend of soil, sand, and organic fertilizer in a 3:1:1 ratio by volume, and cultivated in a greenhouse setting. Following this, both the WT and transgenic plants underwent the previously detailed drought treatment protocol.

### RNA-seq and data analysis

The mature leaves from both well-watered control and drought-treated apple plants were collected at 6 DAT and used for RNA sequencing. For each specimen, triplicate biological libraries were independently assembled and sequenced by Biomarker Technologies Co., Ltd (Beijing, China). Utilizing an Illumina HiSeq 2500 sequencer (Illumina, San Diego, CA), the sequencing process was carried out. The FASTX-toolkit was employed to eliminate adaptor sequences, while FastQC was used to filter out low-quality reads. Subsequently, the clean reads were mapped against the apple genome reference GDDH13 v1.1 [43] with Tophat. Post-alignment, the raw counts of the mapped reads to each gene model were derived and standardized to FPKM. The analysis of differential expression was conducted based on the raw counts and the R package DESeq2 through the BMKCloud platform (www.biocloud.net). DEGs were identified using criteria where the absolute value of log2-fold change was >1 and the false discovery rate (FDR) was <0.01; then these genes were annotated with MapMan (https://mapman.gabipd.org/) as previously described [27].

### RNA extraction and quantitative RT-PCR analysis

Total RNA was extracted via an adapted hexadecyltrimethyl-ammonium bromide (CTAB) technique, followed by DNase treatment to remove any DNA contamination. The RNA was converted into cDNA using the PrimeScript RT Reagent Kit from TaKaRa (Dalian, China), in preparation for subsequent RT-qPCR analysis. The expression levels of target genes were normalized using the housekeeping genes *MdActin* (GenBank: CN938023) and *MdEF-1α* (GenBank: DQ341381) as references. Samples were run in three biological replicates, and the relative expression level of gene was calculated using the delta–delta cycle threshold (ddCT) method. Primers used in this study are listed in Table S1.

### Measurements of soluble sugar and leaf water potential

Soluble sugar was sequentially extracted and derivatized with methoxyamine hydrochloride followed by N-methyl-N-trimethylsilyl-trifluoroacetamide, as detailed in prior studies [44, 45]. The resulting metabolites were then subsequently quantified using a GCMS-2010SE system (Shimadzu, Kyoto, Japan).

Water potential of apple leaves was assayed in a pressure chamber (PMS, Corvallis, OR, USA), following procedure outlined by Song et al. [46].^.^ Briefly, the leaves were removed at their base, leaving 2 cm of the petiole. Nitrogen pressure was used to collect the sap exudate for further analysis.

### Measurements of photosynthetic parameters and chlorophyll fluorescence

The net Pn and Tr were assessed in both normal and drought-treated leaves at intervals of 0, 3, 6, and 9 DAT, utilizing a CIRAS-3 portable photosynthesis instrument (PP systems, Amesbury, MA, USA). Each measurement was carried out using one fully light-exposed and completely expanded mature leaf from each of five plants.

Chlorophyll fluorescence of normal and drought-stressed leaves was monitored with the Dual PAM 100 system (Heinz Walz, Effeltrich, Germany), following a 20-min dark-adaptation phase. The chlorophyll fluorescence parameters, Fv and Fm, were determined using Fluorcam7 software (PSI, Brno, Czech Republic).

### Evaluation of drought resistance and measurement of antioxidant activity

The percentage electrolyte leakage in leaves under well-watered control and drought conditions was determined as previously described [47]. RWC of leaves was determined using the formula: RWC (%) = (FW−DW)/(TW−DW) × 100; in this equation, FW stands for fresh weight, DW for dry weight, and TW for turgid weight of the leaves. Total chlorophyll was extracted from the samples using acetone, and the contents were measured with a spectrophotometer as previously described [48]. The MDA contents in both well-watered and drought-treated samples were determined with the thiobarbituric acid assay following established protocols [49].

The buildup of ROS, such as hydrogen peroxide (H_2_O_2_) and superoxide anion (O_2_^−^), was assessed through histochemical staining with DAB and NBT, respectively. Briefly, leaves were soaked in DAB or NBT solutions overnight, followed by treatment with an ethanol-glycerol mixture to decolorize the chlorophyll. The quantification of H_2_O_2_ and O_2_^−^ concentrations, along with the enzymatic activities of CAT, POD, and SOD, were determined using commercial assay kits sourced from Comin Biotechnology Co., Ltd., Suzhou, China, strictly adhering to the provided guidelines.

In addition, the ROS levels in leaves were also determined by 2′,7′-dichlorodihydrofluorescein diacetate (H_2_DCFDA)-mediated fluorescence detection. Briefly, the leaves of apple plants that had been subjected to drought conditions for six days, as well as those that had been well-hydrated, was trimmed into 1 cm^2^ sections. Then, these leaf segments were immersed in a solution containing 20 mM H_2_DCFDA dissolved in 10 mM Tris–HCl buffer at a pH level of 7.2. This incubation process was conducted in the absence of light for a duration of 30 minutes. Afterward, the leaf pieces were washed with loading buffer to remove the excess dye and were observed using a Zeiss LSM710 laser-scanning confocal microscope at excitation wavelengths of 488 nm for ROS fluorescence [47]. The fluorescence intensity was quantified by Image J software according to the manufacturer’s instructions.

### Measurements of stomatal parameters

At 6 DAT, the functional leaves were sampled to analyze the stomatal morphological parameters as per previous description [46]. Briefly, the leaves were cut into a 1-cm^2^ area then immersed in 100 mM phosphate-buffered saline solution supplemented with 4% glutaraldehyde, and refrigerated at 4°C for 12 h. Images were captured and examined using a scanning electron microscope (SU5000; Hitachi, Tokyo, Japan). The stomatal density and aperture were determined with Image J software.

### Determination of ABA content

The extraction and quantification of ABA were carried out using a liquid chromatography–mass spectrometry (LC–MS) system (QTRAP5500, AB, USA), following established methods [50]. Initially, 100 mg of frozen leaves was placed into a 2-ml centrifuge tube, followed by addition of 1 ml solvent mixture of isopropanol/methanol (80%/20%, v/v), supplemented with 1% glacial acetic acid. After centrifugation at 4°C and 13 000 × g for 10 min, the supernatant was extracted and passed through a 0.22-μm filter. The samples were then measured and analyzed through a InertSustain AQ-C18 column, with a flow rate set at 0.5 ml/min.

### Validation of transcription factor-DNA interactions

In the LUC assay, the promoters of tonoplast-localized sugar transporter genes were cloned into the pGreenII0800-LUC reporter plasmid; meanwhile, the coding sequence of *MdDREB2A* was incorporated into the pGreenII62-SK effector plasmid. These plasmids were then introduced into the *Agrobacterium tumefaciens* strain GV3101 and co-infiltrated into the leaves of 4-week-old *Nicotiana benthamiana* plants. The luciferase activity was measured in triplicate using an Infinite M200 (Tecan, Zürich, Switzerland) three days after transformation.

For the Y1H assay, the full-length promoters of *MdERDL6–1/−2* and *MdTST1/2* were integrated into the pAbAi plasmid; concurrently, the *MdDREB2A* coding sequence was inserted into the pGADT7 plasmid. These plasmids were then transformed and co-expressed in Y1H yeast strains, with selection performed on multiple AbA concentrations.

### Statistical analysis

All data analyses were performed in IBM SPSS Statistics 23 and graphs visualized with Sigma Plot 12.5 software. Data was statistically assessed using independent *t* tests or one-way ANOVA, and statistical significance inferred at *P* < 0.05.

## Supplementary Material

Web_Material_uhae251

## Data Availability

The data underlying this article are available in the article and in its online supplementary material.
