# Supplementary material for: Apple vacuolar sugar transporters regulated by MdDREB2A enhance drought resistance by promoting accumulation of soluble sugars and activating ABA signaling
Source: Hortic Res. 2024 Sep 3;11(12):uhae251. doi: 10.1093/hr/uhae251 (PMC11630069; doi:10.1093/hr/uhae251)
Supplement: Web_Material_uhae251 [file web_material_uhae251.zip › Supplementary Information.pdf]

Supplementary Information

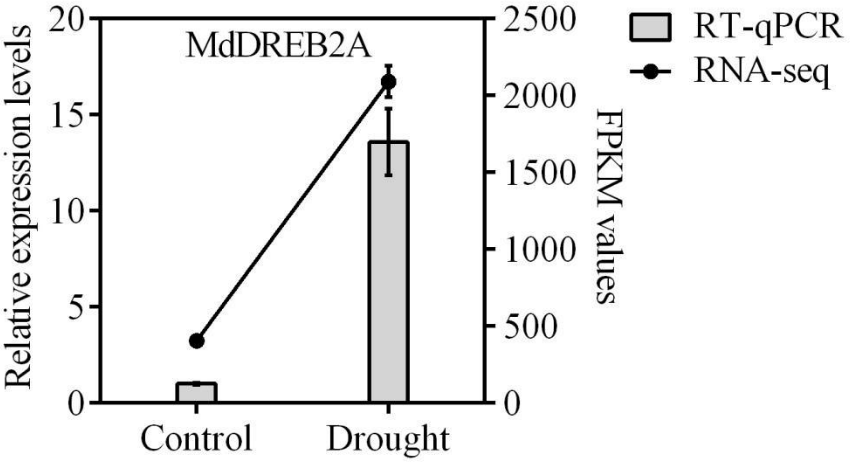

Figure S1. Relative expression levels based on RT-qPCR and FPKM values based on RNA-seq of *MdDREB2A* in well-watered control and drought-treated apple leaves.

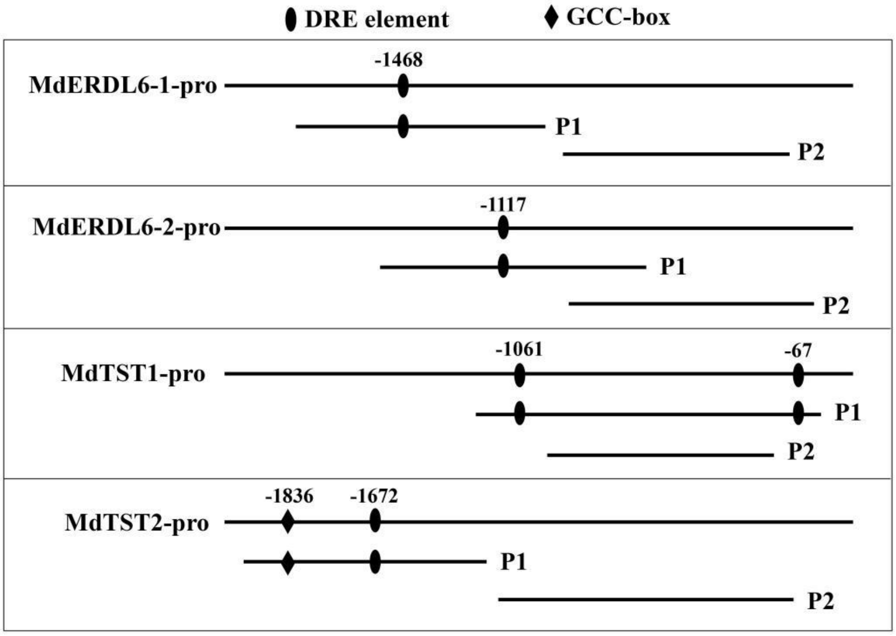

Figure S2. Schematic diagram of DRE and GCC-box elements distribution in promoters of *MdERDL6-1/-2* and *MdTST1/2*. The 2 kb of promoters were retrieved from GDR website (https://www.rosaceae.org/) respectively, and the *cis*-elements were analyzed by PLANTCARE (http://bioinformatics.psb.ugent.be/webtools/plantcare/html/). The P1 and P2 represent the truncated promoters with and without DRE and GCC-box element, respectively.

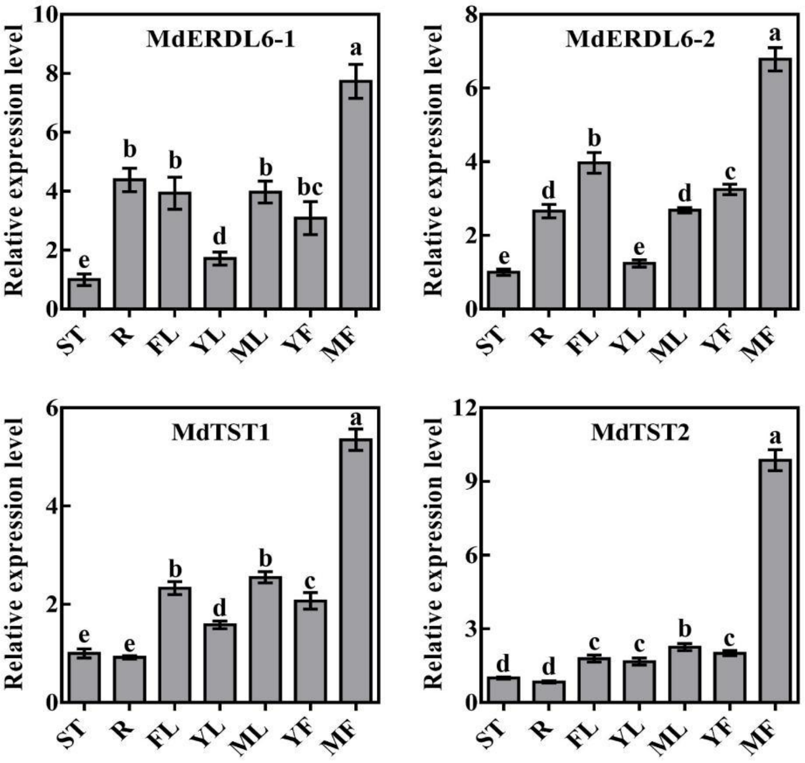

Figure S3. The expression profiles of *MdERDL6-1/-2* and *MdTST1/2* in apple tissues. The transcript levels were normalized with those of *MdActin* and *MdEF-1α*. Relative expression levels for each gene were obtained via ddCT method, with the expression in ST set as 1. Bars represent the mean value ± SD (n = 3). Different letters indicate significant differences at $P < 0.05$. ST, shoot tips; R, root; FL, flowers; YL, young leaves; ML, mature leaves; YF, young fruits; MF, mature fruits.

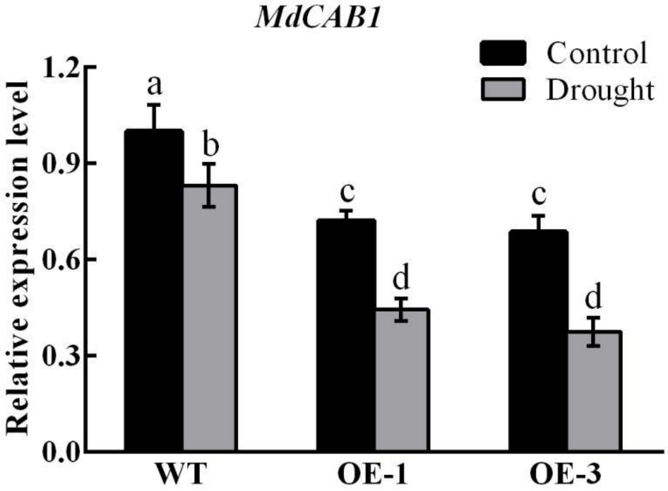

Figure S4. The mRNA expression of *MdCAB1* in the transgenic apple (OE-1 and OE-3) overexpressing *MdERDL6-1* under drought condition. The transcript levels were normalized with those of *MdActin* and *MdEF-1α* in transgenic apple. Relative expression levels for each gene were obtained via ddCT method, with the expression in WT set as 1. WT, wild type. Bars represent the mean value ± SD (n = 3). Different letters indicate significant differences at $P < 0.05$.

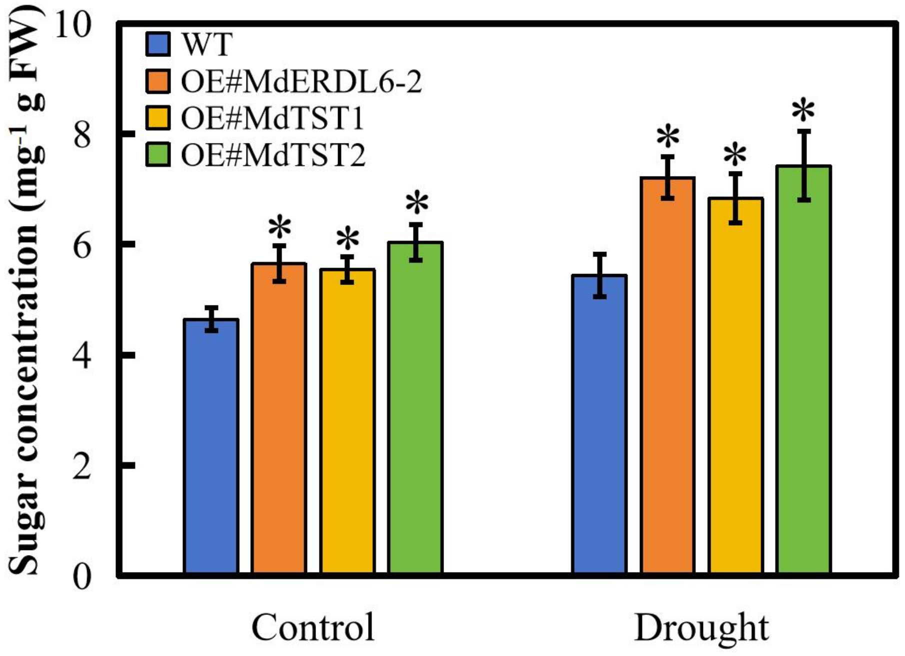

Figure S5. Sugar concentrations in the leaves of wild-type and overexpression of *MdERDL6-2*, *MdTST1*, and *MdTST2 Arabidopsis* under well-watered control and drought treatment. WT, wild-type; FW, fresh weight. The bars represent the mean value ± SD (n = 3). **P* < 0.05, a significant difference from WT.

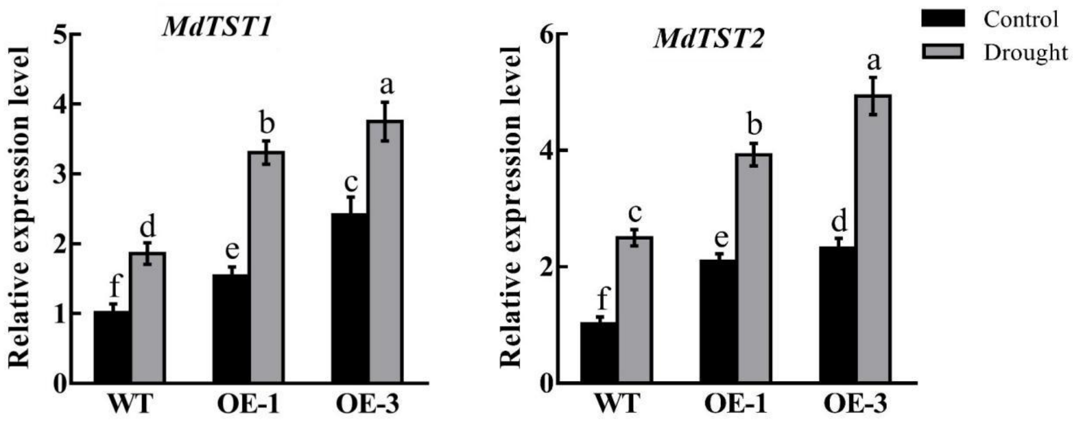

Figure S6. The mRNA expression of sugar transporter genes *MdTST1* and *MdTST2* in the transgenic apples overexpressing *MdERDL6-1* under drought condition. The transcript levels were normalized with those of *MdActin* and *MdEF-1α*. Relative expression levels for each gene were obtained via ddCT method, with the expression in WT of well-watered condition set as 1. WT, wild type. Bars represent the mean value ± SD (n = 3). Different letters indicate significant differences at $P < 0.05$.

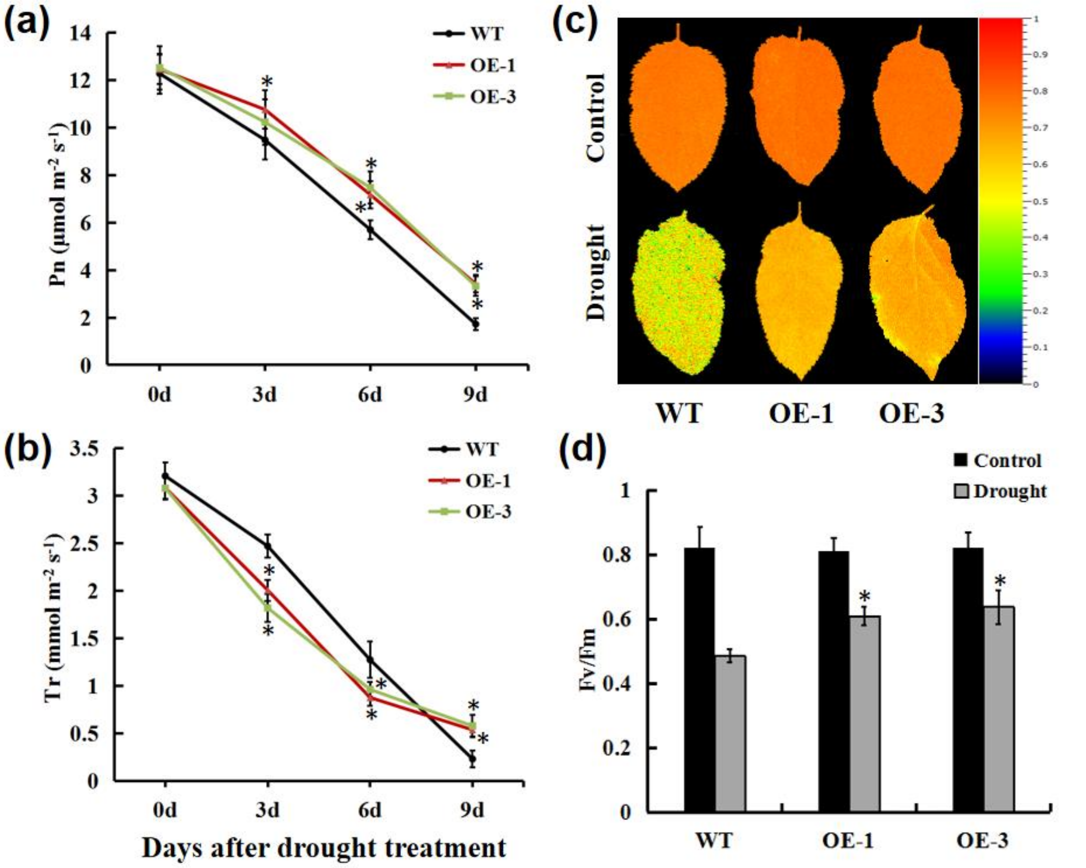

Figure S7. Effects of *MdERDL6-1* overexpression in apple on the photosynthetic and chlorophyll fluorescence parameters under drought condition. **(a-b)** Changes of the net photosynthesis rates (Pn) and transpiration rates (Tr) in WT and *MdERDL6-1*-overexpressing apple leaves during drought stress treatment. **(c-d)** Chlorophyll fluorescence images and Fv/Fm ratios in WT and *MdERDL6-1*-overexpressing apple leaves after 9 days under normal or drought condition. WT, wild type. The bars represent the mean value ± SD (n = 3). *$P < 0.05$, a significant difference from control.

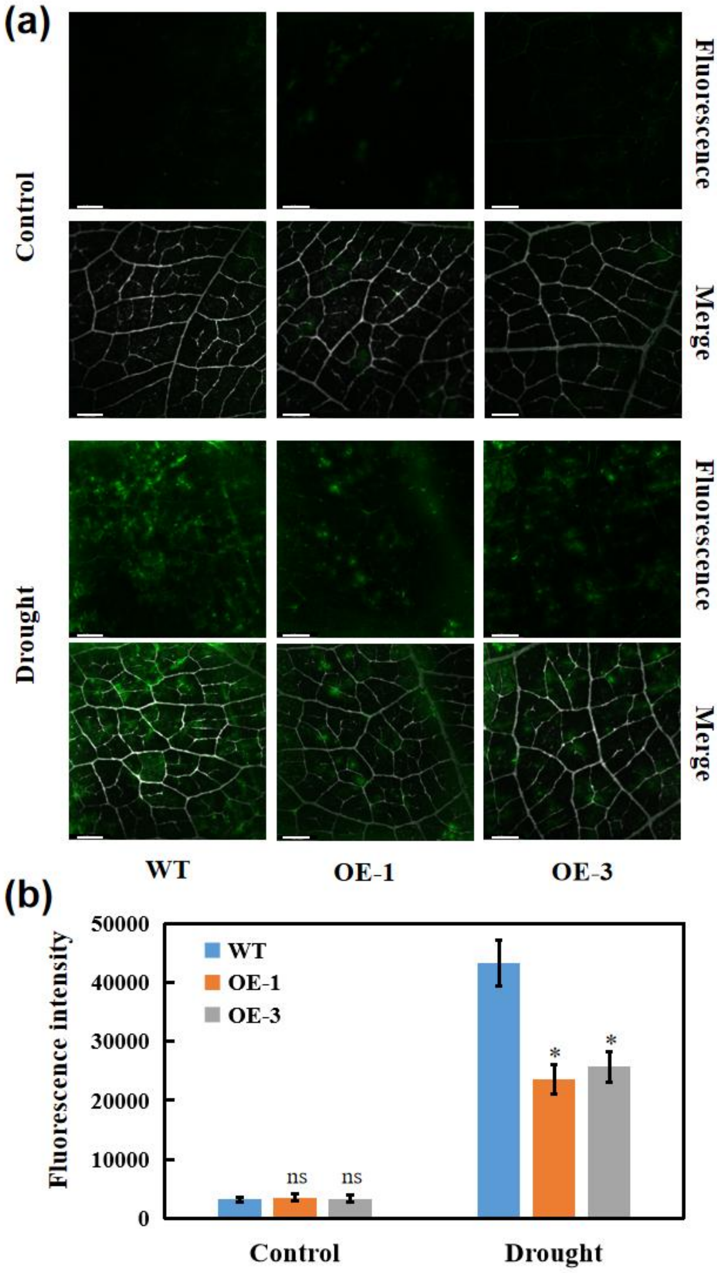

Figure S8. Changes of ROS concentrations in leaves of the WT and *MdERDL6-1*-overexpressing apple plants under normal and drought stress. **(a)** Fluorescence detection of ROS accumulation in leaves of WT and *MdERDL6-1*-overexpressing apple plants using H_2DCFDA under normal and drought stress. Scale bar = 1 mm. **(b)** Fluorescence intensity was determined to assess the ROS concentrations. WT, wild type. The bars represent the mean value ± SD (n = 3). *$P < 0.05$, a significant difference from WT.

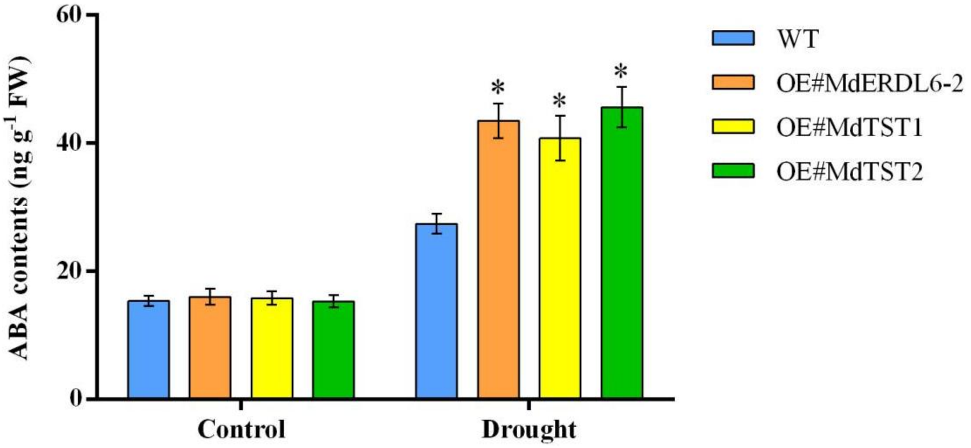

Figure S9. ABA contents in the WT and *MdERDL6-2/MdTST1/MdTST2* overexpressing *Arabidopsis* under normal and drought stress. The bars represent mean values ± SD (n = 3). *P < 0.05 indicate significant difference relative to the WT treatment. WT, wild type.

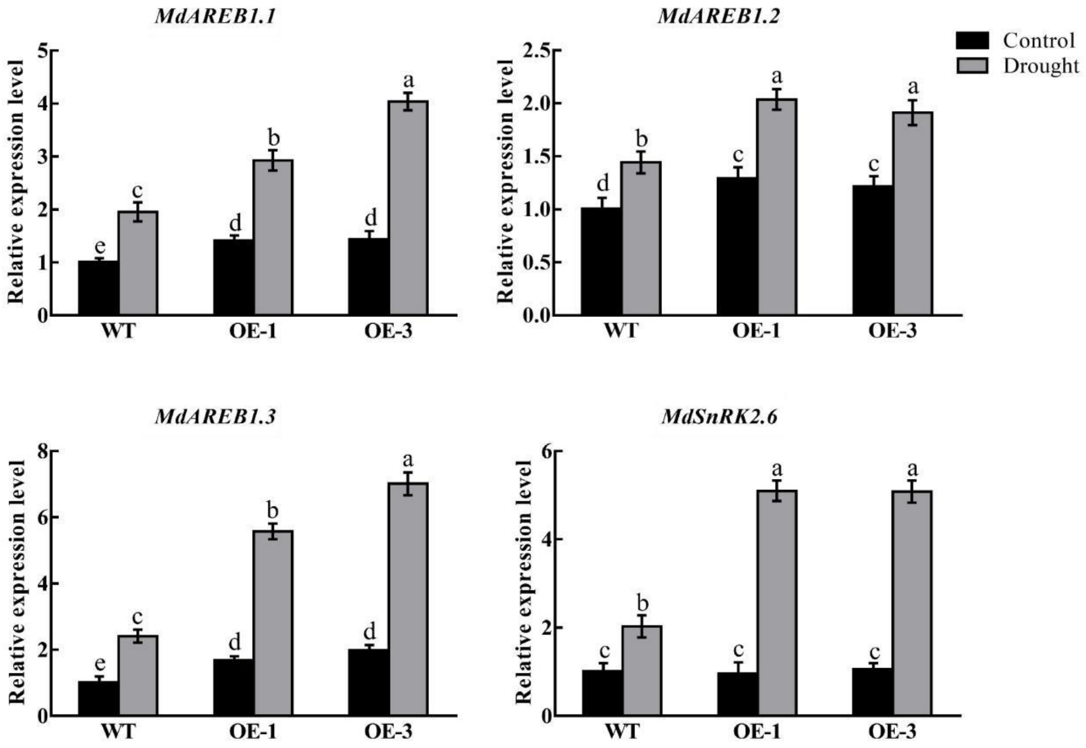

Figure S10. The mRNA expression of genes related to drought stress response in the transgenic apples overexpressing *MdERDL6-1* under drought condition. The transcript levels were normalized with those of *MdActin* and *MdEF-1α*. Relative expression levels for each gene were obtained via ddCT method, with the expression in WT of well-watered condition set as 1. WT, wild type. Bars represent the mean value ± SD (n = 3). Different letters indicate significant differences at $P < 0.05$.

Table S1 Primers used in this study

Primer name	Sequence (5`-3`)
qMdERDL6-1	F:GGCATCATATCAGTGGTTGG<br>R:AGGTTCCTCCACTGCTCCAT
qMdCAB1	F:ATCTTGATGGGTGCCGTGGAG<br>R:AGCCTCTGTGTCTTCTGCAAGG
qMdTST1	F:TCGTCTATTTCTGCGTCTTTGTC<br>R:CCGCTGCGTAAATCCCAAAT
qMdTST2	F:GTACCGAACGATGGTCAGTTCTTC<br>R:TGACTCCGGGTTCGAAAAGGTC
qMdActin	F:GGCTGGATTTGCTGGTGATG<br>R:TGCTCACTATGCCGTGCTCA
qMdEF-1α	F:ATTCAAGTATGCCTGGGTGC<br>R:CAGTCAGCCTGTGATGTTCC
qMdAREB1.1	F:GTGCCGGCGGGTTTAATATG<br>R:CAATCCCGCGATTCAGTTGC
qMdAREB1.2	F:AAGCAGGCTTACACAACGGA<br>R:AGCATCGCTTCTTTCCTCGT
qMdAREB1.3	F:CGTACGATCCACTGAGGTTTAAC<br>R:GAAAGCTAGCTGTGCAAACAACTC
qMdSnRK2.6	F:TTGGATTCACGAGCTGTAGGA<br>R:TGATGGGAAAAGGTGGTGGG
qMdNCED1	F: TCGTCCAAGAAAGGGAAATG<br>R: GTGTGCGATCATTGTTGACC
qMdNCED3	F: TCCCCCTCTTCTTCCTCAAT<br>R: GTTCCATTGTTGTGGCACTG
MdDREB2A-CDS	F:ATGGGAGCTTATGATCAAGG<br>R:CATTTCCATCGAATAGTTGT
MdERDL6-1-pro	F:ACTATCTAGTAGCTATGTGGGTTG<br>R:TCGACGAAATCCCCCAACG
MdERDL6-1-P1	F:GTCCCATATTGACAAAGTT<br>R:CACATATCATTGTCCGTCCG
MdERDL6-1-P2	F:CCACTGGCCTACAACTAC<br>R:AGGAGTCGAGAGTTACCA
MdERDL6-2pro	F:GAAGGAGTTCATTAGCTGGA<br>R:ATACGTCCAACTGCGATCCT
MdERDL6-2-P1	F:TCAAAGAGAAACATTAGTTA<br>R:CAACCGCAATGGAAATAGG
MdERDL6-2-P2	F:AGACAGTAATTTGGTAACTA

Primer name	Sequence (5`-3`)
	R:TGGAAAGGGATATCTTTGATCC
MdTST1pro	F:TCCCTAGAACTTGGTGGGAGAG<br>R:AAGAGAAACAGAGGGACAAAGAGAT
MdTST1-P1	F:ACCCAACGCAGCATCATC<br>R:GTTCGGACTTTTTCTCCGAT
MdTST1-P2	F:GCTTGGTAGATTTTAGGGTC<br>R:GGACAAACAATGACAGATGC
MdTST2pro	F:ACATCTTGCATTTTTAGACGCATGT<br>R:AACCCAGCTCAGCTCTTGAATATTT
MdTST2-P1	F:TCTCCTACTCCCACACACAC<br>R:GGCCACTTAGAGCCTTCACT
MdTST2-P2	F:CAACTTGTTCTGACCTAGTA<br>R:ATTGCTGAGACTTAGTCTACTT
